# Supplementary material for: A TRPV4-dependent calcium signaling axis governs lamellipodial actin architecture to promote cell migration
Source: bioRxiv. 2025 Mar 30:2025.03.28.646012. Preprint. [Version 1] doi: 10.1101/2025.03.28.646012 (PMC11974816; doi:10.1101/2025.03.28.646012)
Supplement: Supplement 1 [file NIHPP2025.03.28.646012v1-supplement-1.pdf]

bioRxiv preprint doi: https://doi.org/10.1101/2025.03.28.646012; this version posted March 30, 2025. The copyright holder for this preprint (which was not certified by peer review) is the author/funder, who has granted bioRxiv a license to display the preprint in perpetuity. It is made available under aCC-BY 4.0 International license.

SUPPLEMENTAL FIGURES

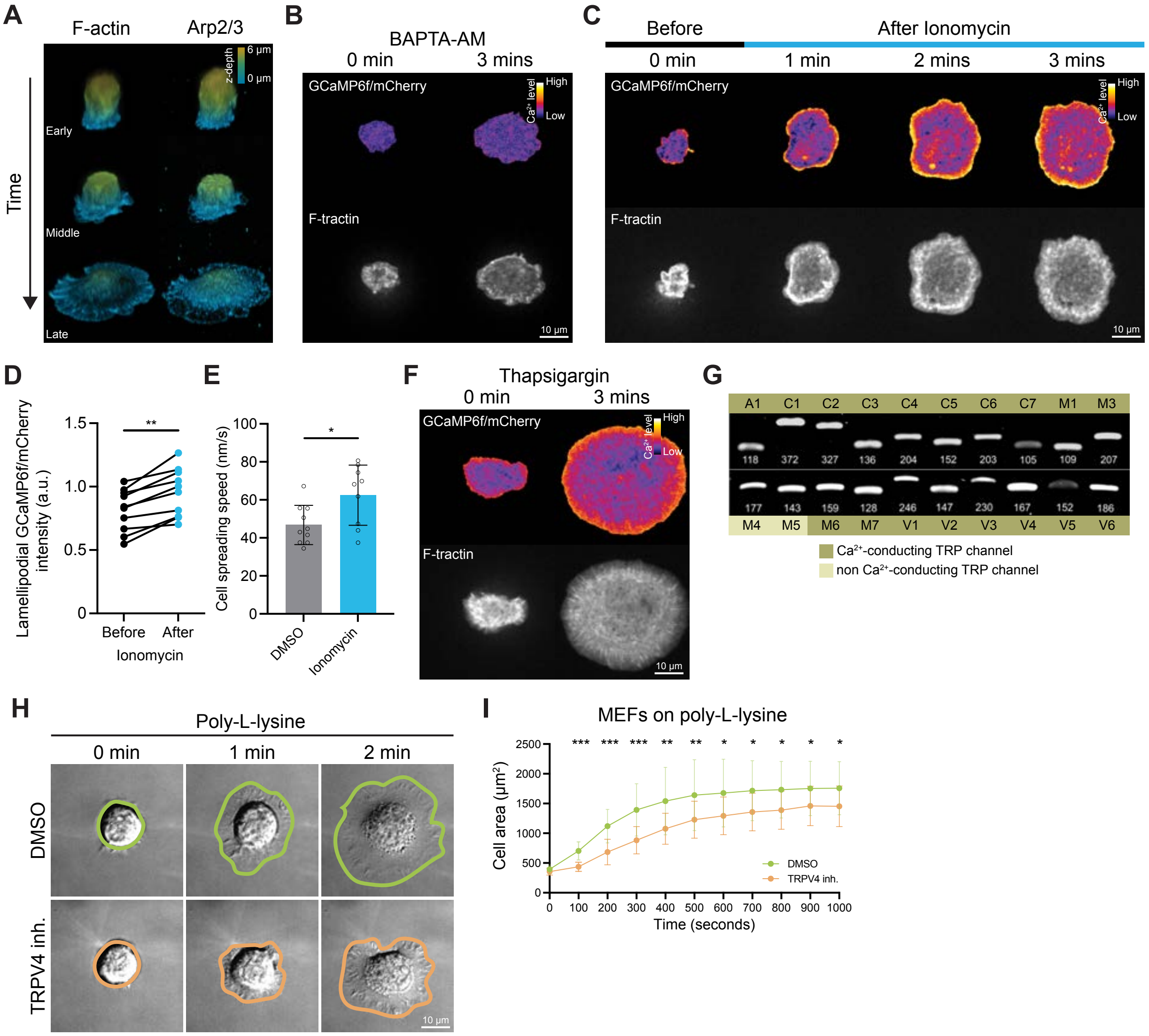

Figure S1. Perturbations in Ca^{2+} signaling, by Ca^{2+} drugs or TRPV4 inhibitor, affects cell spreading independently of integrin signaling. Related to Figure 1.

(A) 3D projection of a spreading MEF in 3 stages (early, middle and late). (Left) Fluorescent phalloidin and (Right) anti-ArpC2 staining. The images are color-coded based on the z-depth.

(B-F) Effects of Ca^{2+} ionophore, chelator and endoplasmic reticulum Ca^{2+} ATPases (SERCA pumps) inhibitor on cell spreading. Timelapse ratiometric GCaMP6f/mCherry and F-tractin-emiRFP670 images of a spreading cell treated with (B) 10 µM BAPTA-AM and (C) 1 µM ionomycin. Quantifications of (D) the lamellipodial GCaMP6f/mCherry intensity and (E) the average cell spreading speed before and after the addition of ionomycin. n ≥ 9 cells per condition. (F) Timelapse ratiometric GCaMP6f/mCherry and F-tractin-emiRFP670 images of a spreading cell treated with 1 µM thapsigargin.

bioRxiv preprint doi: https://doi.org/10.1101/2025.03.28.646012; this version posted March 30, 2025. The copyright holder for this preprint (which was not certified by peer review) is the author/funder, who has granted bioRxiv a license to display the preprint in perpetuity. It is made available under aCC-BY 4.0 International license.

(G) Reverse transcription PCR (RT-PCR) analysis of TRP family channels expressed in MEFs. The TRP channels that are labelled in dark and light green are Ca^{2+}-conducting and non Ca^{2+}-conducting, respectively.

(H-I) Effects of poly-L-lysine coated substrate on cell spreading. (H) DIC timelapse images of spreading MEFs treated with (top) DMSO and (bottom) TRPV4 inhibitor. The colored outline shows the cell boundary. (I) Quantifications of cell spread area over time in DMSO and TRPV4 inhibitor conditions. n = 20 cells per condition.

Mann-Whitney test is used for comparisons between 2 treatments and one-way ANOVA followed by Tukey-Kramer post-hoc test for comparisons between 3 or more treatments. The data are presented as mean ± standard deviation (SD). * $p < 0.05$, ** $p < 0.01$, *** $p < 0.005$, **** $p < 0.001$.

bioRxiv preprint doi: https://doi.org/10.1101/2025.03.28.646012; this version posted March 30, 2025. The copyright holder for this preprint (which was not certified by peer review) is the author/funder, who has granted bioRxiv a license to display the preprint in perpetuity. It is made available under aCC-BY 4.0 International license.

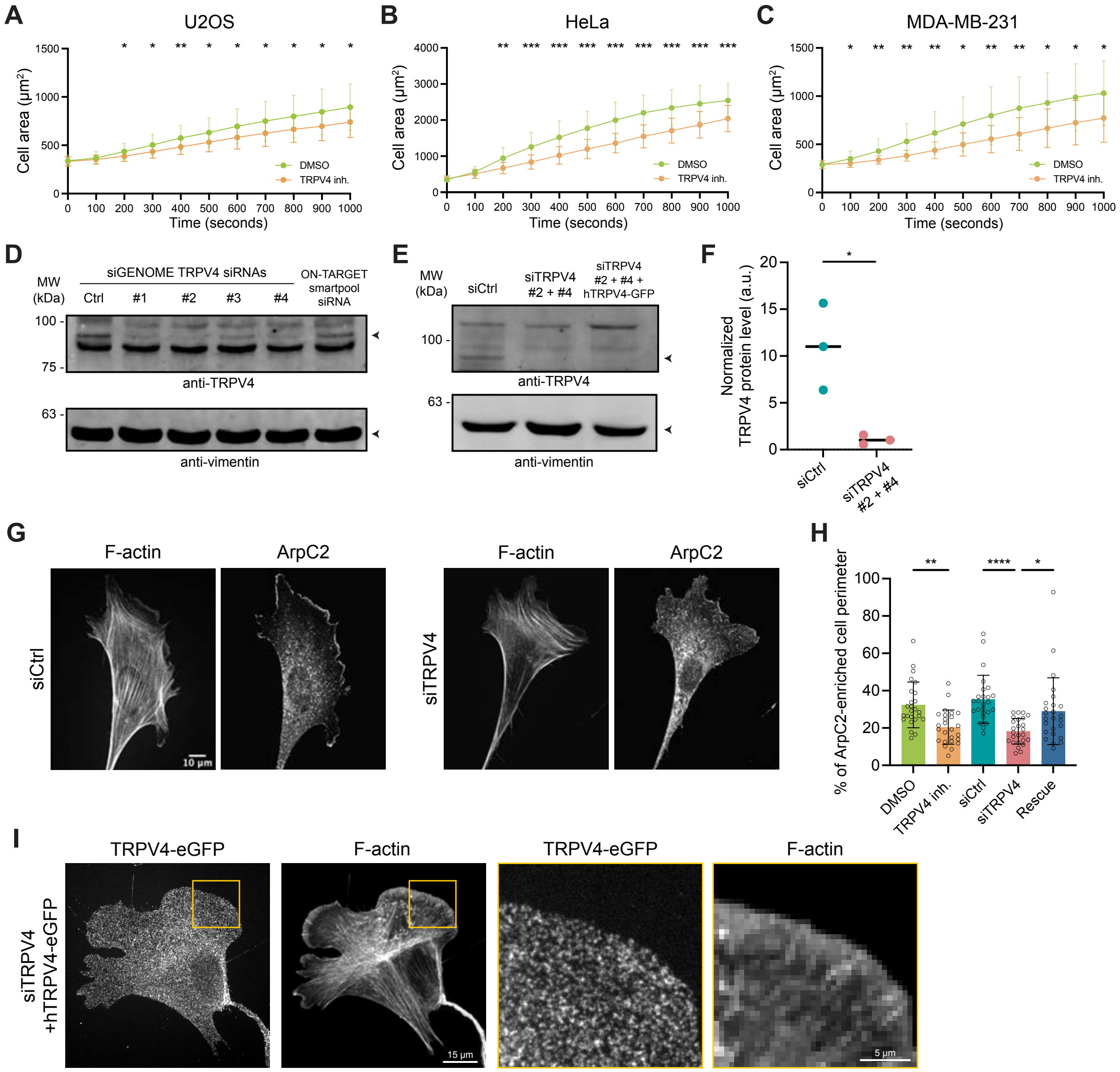

Figure S2. Suppression of TRPV4 impedes cell spreading across different cell types and disrupts lamellipodia formation. Related to Figure 1.

(A-C) Quantifications of (A) U2OS, (B) HeLa and (C) MDA-MB-231 cell spread area over time in the presence of DMSO or TRPV4 inhibitor. n = 20 cells per condition.

(D-F) Western blot analysis of endogenous TRPV4 level in siCtrl, siTRPV4, and rescue treatments 24 hr post-transfection. (D) Western blots showing the TRPV4 knockdown efficiencies of individual siRNAs and (E) a combination of TRPV4 siRNAs #2 and #4. (F) Quantifications of TRPV4 knockdown efficiency. n = 3 biological repeats.

(G-H) Analysis of percent of ArpC2-enriched cell edge upon TRPV4 suppression. (G) Fluorescent phalloidin and anti-ArpC2 images of cells treated with 0.1% DMSO and TRPV4 inhibitor. (H) Quantifications of the percent of ArpC2-enriched cell edge. n > 20 cells per condition.

bioRxiv preprint doi: https://doi.org/10.1101/2025.03.28.646012; this version posted March 30, 2025. The copyright holder for this preprint (which was not certified by peer review) is the author/funder, who has granted bioRxiv a license to display the preprint in perpetuity. It is made available under aCC-BY 4.0 International license.

(I) Immunofluorescence images of a TRPV4 siRNA treated MEF expressing hTRPV4-eGFP. The cell was stained for phalloidin (F-actin) and anti-GFP (hTRPV4-eGFP).

Mann-Whitney test is used for comparisons between 2 treatments and one-way ANOVA followed by Tukey-Kramer post-hoc test for comparisons between 3 or more treatments. The data are presented as mean ± SD unless otherwise specified. * $p < 0.05$, ** $p < 0.01$, *** $p < 0.005$, **** $p < 0.001$.

bioRxiv preprint doi: https://doi.org/10.1101/2025.03.28.646012; this version posted March 30, 2025. The copyright holder for this preprint (which was not certified by peer review) is the author/funder, who has granted bioRxiv a license to display the preprint in perpetuity. It is made available under aCC-BY 4.0 International license.

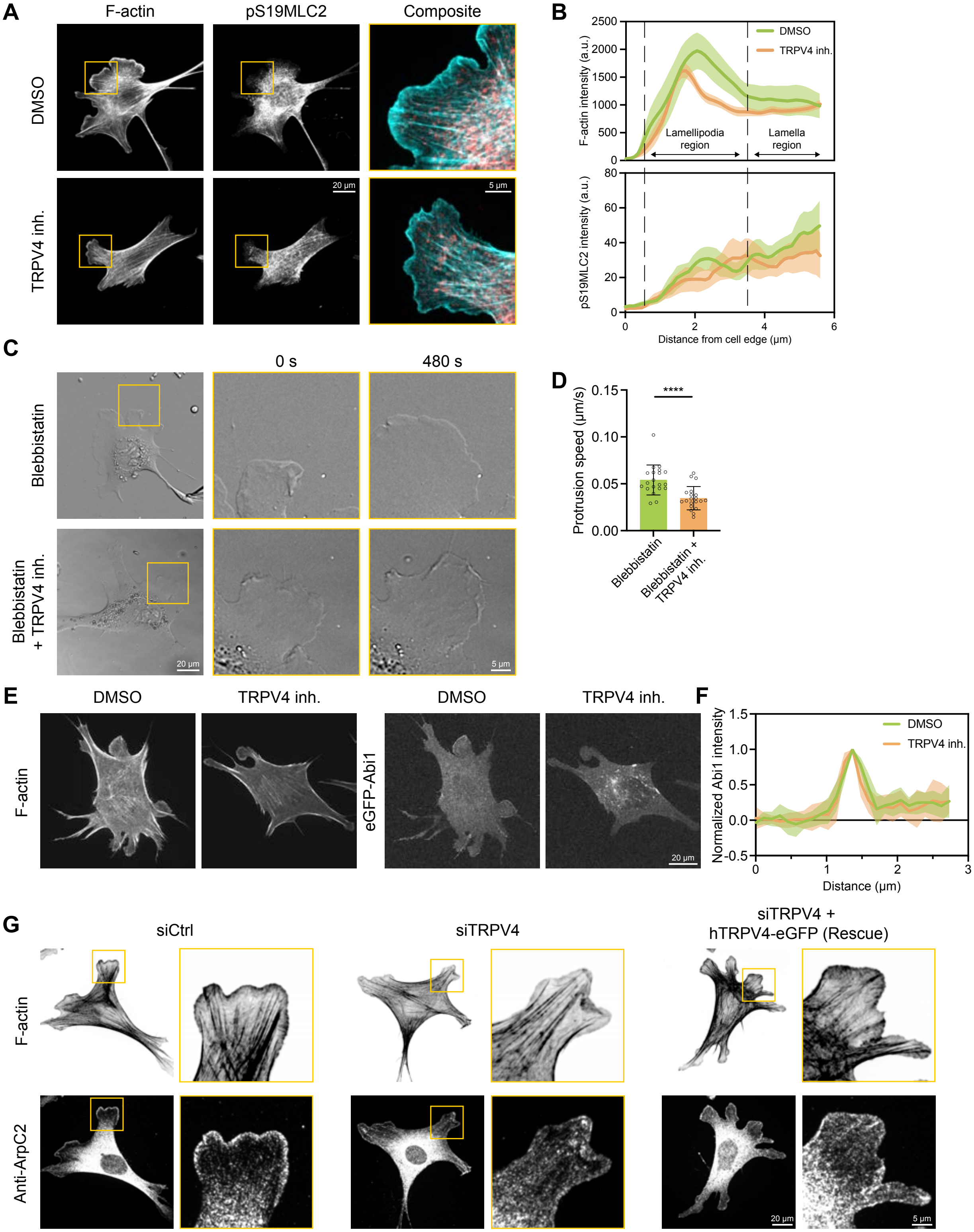

Figure S3. Myosin-II and the upstream regulators of Arp2/3 are not involved in the control of lamellipodia protrusive activity by TRPV4. Related to Figure 3.

bioRxiv preprint doi: https://doi.org/10.1101/2025.03.28.646012; this version posted March 30, 2025. The copyright holder for this preprint (which was not certified by peer review) is the author/funder, who has granted bioRxiv a license to display the preprint in perpetuity. It is made available under aCC-BY 4.0 International license.

(A-B) Analysis of the effects of TRPV4 inhibition on phospho-myosin light chain (pS19MLC2). (A) F-actin and phospho-myosin light chain (pS19MLC2) images of MEFs treated with 0.1% DMSO and TRPV4 inhibitor (10 µM HC-067047). (B) Average line intensity profiles of the (top) F-actin and (bottom) pS19MLC2 staining near the cell edge in the presence of DMSO or TRPV4 inhibitor. $n \geq 6$ cells per condition.

(C-D) Analysis of the effects of myosin-II inhibition on lamellipodia protrusion speed. (C) DIC images of (left) blebbistatin-treated and (right) TRPV4 inhibitor-and-blebbistatin-co-treated MEFs. The yellow box indicates the region of the zoom-in shown on the right. (D) Quantifications of their protrusion speed. $n = 20$ cells per condition.

(E-F) Localization of Abi1 in TRPV4 inhibited MEFs. (E) Fluorescent images of (left) F F-tractin-emiRFP670 and (right) eGFP-Abi1. (F) Average line intensity profiles of eGFP-Abi1 near the cell edge in the presence of DMSO or TRPV4 inhibitor. $n = 9$ cells per condition.

(G) Fluorescent phalloidin and anti-ArpC2 images of MEFs treated with non-targeting siRNA (siCtrl), TRPV4 siRNA and TRPV4 siRNA + human TRPV4-eGFP (siRNA-resistant).

Mann-Whitney test is used for comparisons between 2 treatments and one-way ANOVA followed by Tukey-Kramer post-hoc test for comparisons between 3 or more treatments. The data are presented as mean ± SD unless otherwise specified. * $p < 0.05$, ** $p < 0.01$, *** $p < 0.005$, **** $p < 0.001$.

bioRxiv preprint doi: https://doi.org/10.1101/2025.03.28.646012; this version posted March 30, 2025. The copyright holder for this preprint (which was not certified by peer review) is the author/funder, who has granted bioRxiv a license to display the preprint in perpetuity. It is made available under aCC-BY 4.0 International license.

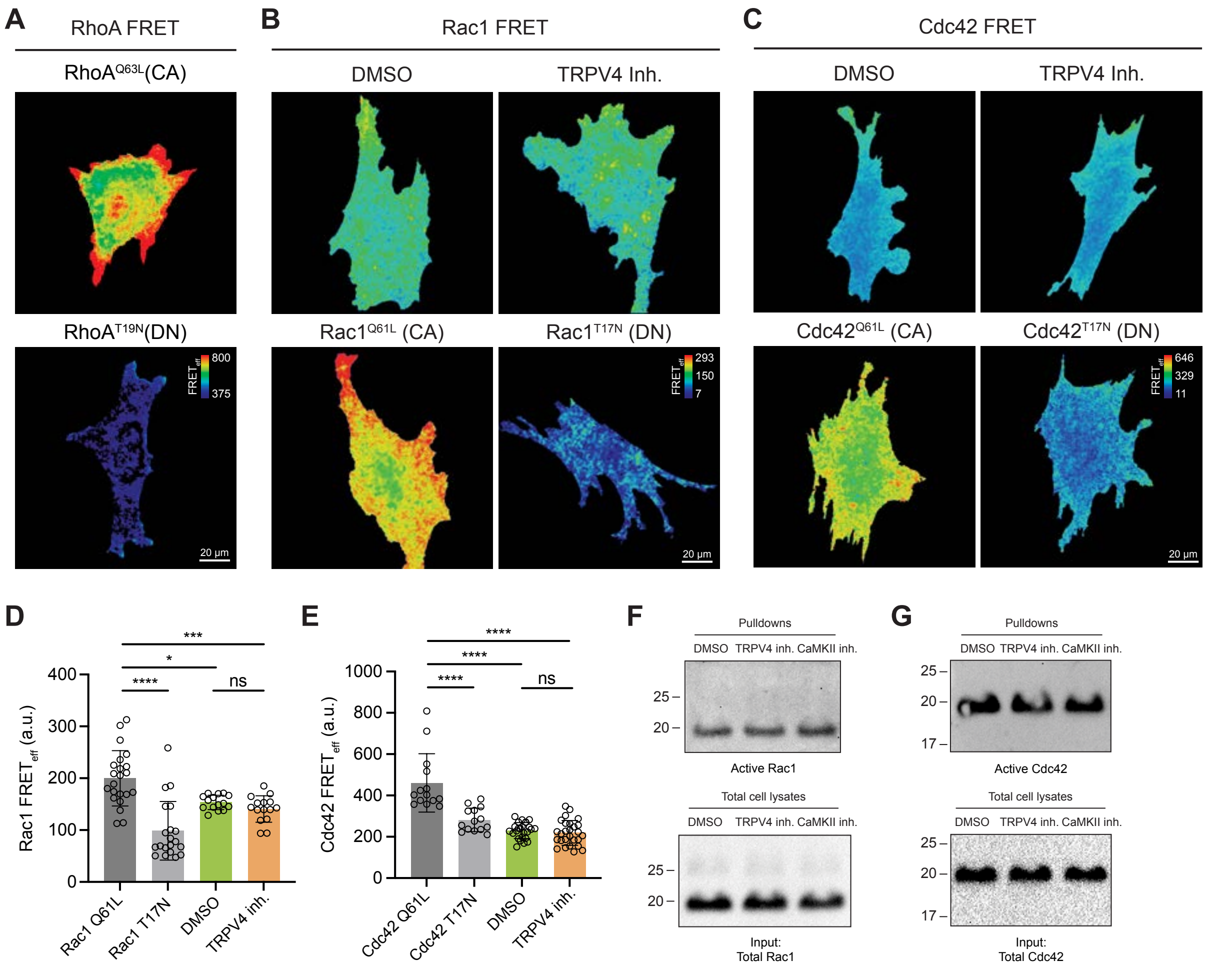

Figure S4. TRPV4 does not regulate the activity of Rac1 or Cdc42. Related to Figure 4.

(A) Fluorescence images of RhoA FRET biosensor activation in MEFs expressing constitutively active (CA) or dominant negative (DN) RhoA mutant biosensors (B-E) Analyses of the effects of TRPV4 inhibition on Rac1 and Cdc42 activity by Förster Resonance Energy Transfer (FRET) imaging. MEFs were transiently expressing a Rac1 or Cdc42 FRET biosensor. Fluorescence images of (B) Rac1 and (C) Cdc42 FRET biosensors activation in cells treated with 0.1% DMSO or 10 µM HC-067047. Quantifications of the global (D) Rac1 and (E) Cdc42 $FRET_{eff}$. $n \geq 14$ cells per condition.

(F-G) Western blot analysis of the activity of (F) Rac1 and (G) Cdc42 in MEFs treated with 0.1% DMSO, TRPV4 inhibitor (10 µM HC-067047), or CaMKII inhibitor (10 µM KN-93) by using the active GTPase pull-down assay.

Mann-Whitney test is used for comparisons between 2 treatments and one-way ANOVA followed by Tukey-Kramer post-hoc test for comparisons between 3 or more treatments. The data are presented as mean ± SD unless otherwise specified. * $p < 0.05$, ** $p < 0.01$, *** $p < 0.005$, **** $p < 0.001$.

bioRxiv preprint doi: https://doi.org/10.1101/2025.03.28.646012; this version posted March 30, 2025. The copyright holder for this preprint (which was not certified by peer review) is the author/funder, who has granted bioRxiv a license to display the preprint in perpetuity. It is made available under aCC-BY 4.0 International license.

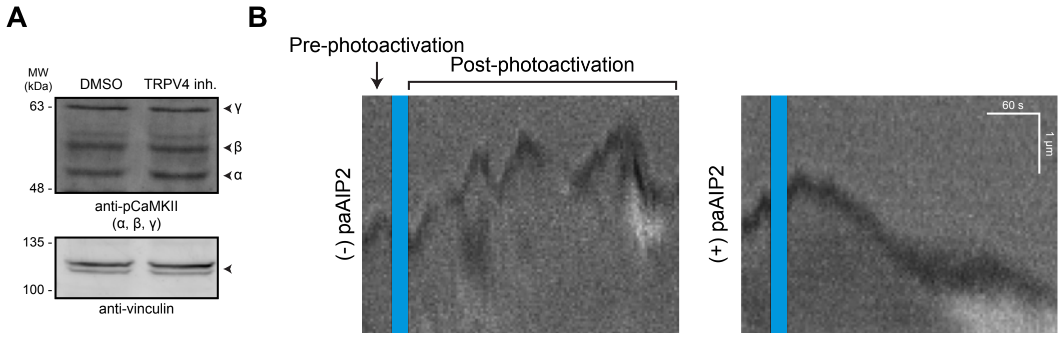

Figure S5. Local suppression of CaMKII perturbs lamellipodia protrusion dynamics. Related to Figure 5.

(A) Western blot analysis of the level of phospho-CaMKII in MEFs treated with 0.1% DMSO or TRPV4 inhibitor (10 µM HC-067047). 3 isoforms of CaMKII (α, β and γ) are expressed. Vinculin was used as a loading control.

(B) DIC Kymographs generated from the cells in the optogenetic CaMKII inhibition experiment. The kymographs show cell edge dynamics before and after photo-activation. The blue stripe indicates the photo-illumination period. They were extracted from cells without paAIP2 or with paAIP2 expression.

bioRxiv preprint doi: https://doi.org/10.1101/2025.03.28.646012; this version posted March 30, 2025. The copyright holder for this preprint (which was not certified by peer review) is the author/funder, who has granted bioRxiv a license to display the preprint in perpetuity. It is made available under aCC-BY 4.0 International license.

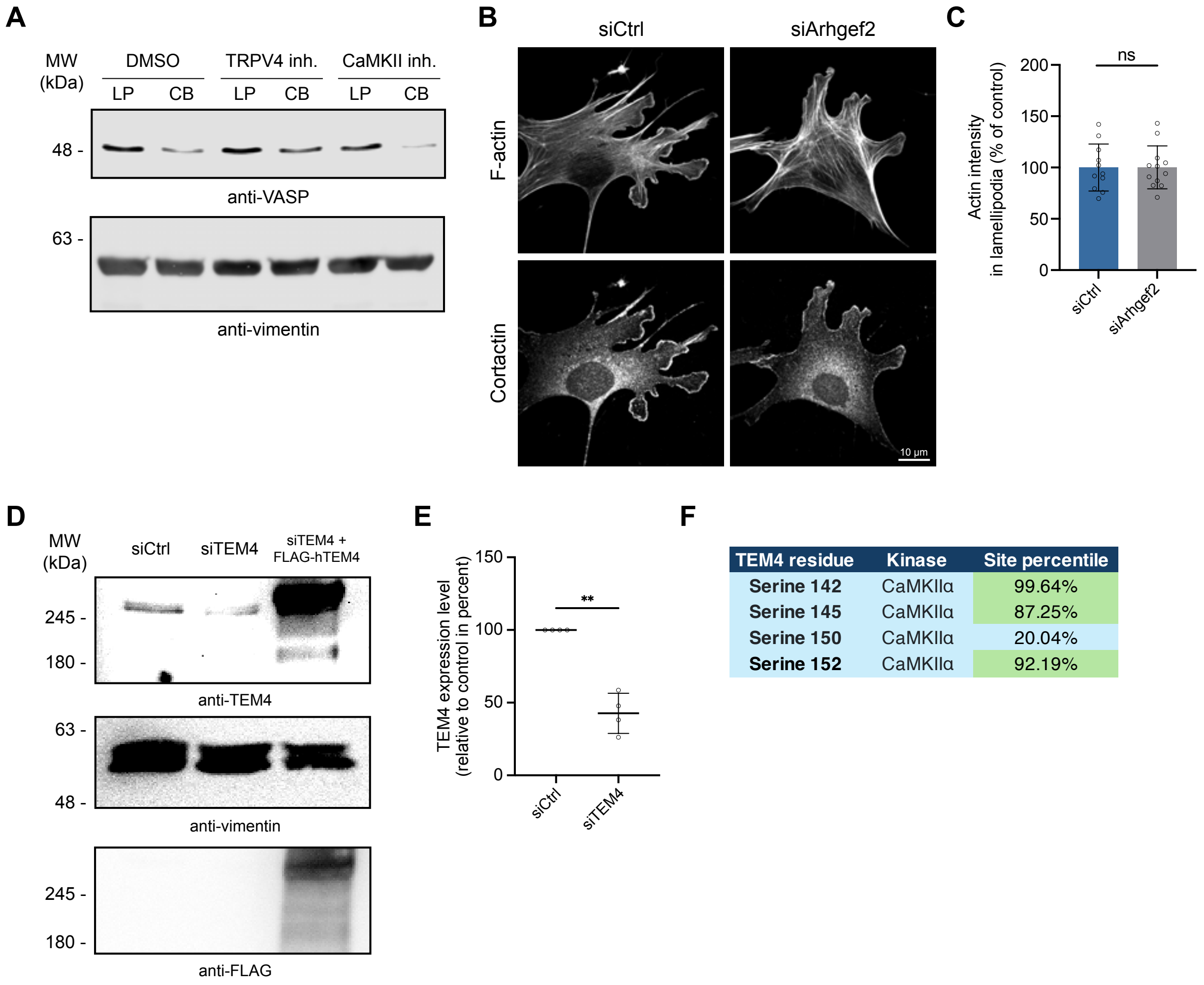

TEM4 residue	Kinase	Site percentile
Serine 142	CaMKIIα	99.64%
Serine 145	CaMKIIα	87.25%
Serine 150	CaMKIIα	20.04%
Serine 152	CaMKIIα	92.19%

Figure S6. Arhgef2/GEF-H1 does not regulate lamellipodial F-actin. Related to Figure 6.

(A) Western blot analysis of VASP (a lamellipodia marker) in purified lamellipodia (LP) and cell body (CB) fractions from MEFs migrating across a 3.0-µm pore size polycarbonate membrane. Prior to the fractionation, MEFs were treated with 0.1% DMSO, TRPV4 inhibitor (10 µM HC-067047), or CaMKII inhibitor (10 µM KN-93). Vimentin was used as a loading control.

(B-C) Analysis of the effects of Arhgef2/GEF-H1 depletion on F-actin. (B) Fluorescent phalloidin and anti-cortactin images of MEFs treated with non-targeting siRNA (siCtrl) and Arhgef2/GEF-H1 siRNA (siArhgef2). (C) Quantifications of lamellipodial F-actin intensity upon Arhgef2 depletion. n = 10 cells per condition.

(D-E) Western blot analysis of endogenous TEM4 level in siCtrl, siTEM4, and rescue treatments 72 hr post-transfection. (D) Western blots showing the TEM4 knockdown efficiency of the smart pool TEM4 siRNA and the over-expression of 3×FLAG-hTEM4 in TEM4-depleted MEFs. Vimentin was used as a loading control. (E) Quantification of TEM4 knockdown efficiency. n = 4 biological repeats.

(F) Kinase prediction analysis of TEM4. The table shows the phosphorylation site percentile of TEM4 at serine residues 142, 145, 150 and 152 by CaMKIIα. Score percentiles that are above 80% were highlighted

bioRxiv preprint doi: https://doi.org/10.1101/2025.03.28.646012; this version posted March 30, 2025. The copyright holder for this preprint (which was not certified by peer review) is the author/funder, who has granted bioRxiv a license to display the preprint in perpetuity. It is made available under aCC-BY 4.0 International license.

in green. The analysis was performed by using the PhosphoSite Plus database (www.phosphosite.org/uniprotAccAction?id=Q96PE2)[98] .

Mann-Whitney test is used for comparisons between 2 treatments. The data are presented as mean ± SD unless otherwise specified. * $p < 0.05$, ** $p < 0.01$, *** $p < 0.005$, **** $p < 0.001$.

bioRxiv preprint doi: https://doi.org/10.1101/2025.03.28.646012; this version posted March 30, 2025. The copyright holder for this preprint (which was not certified by peer review) is the author/funder, who has granted bioRxiv a license to display the preprint in perpetuity. It is made available under aCC-BY 4.0 International license.

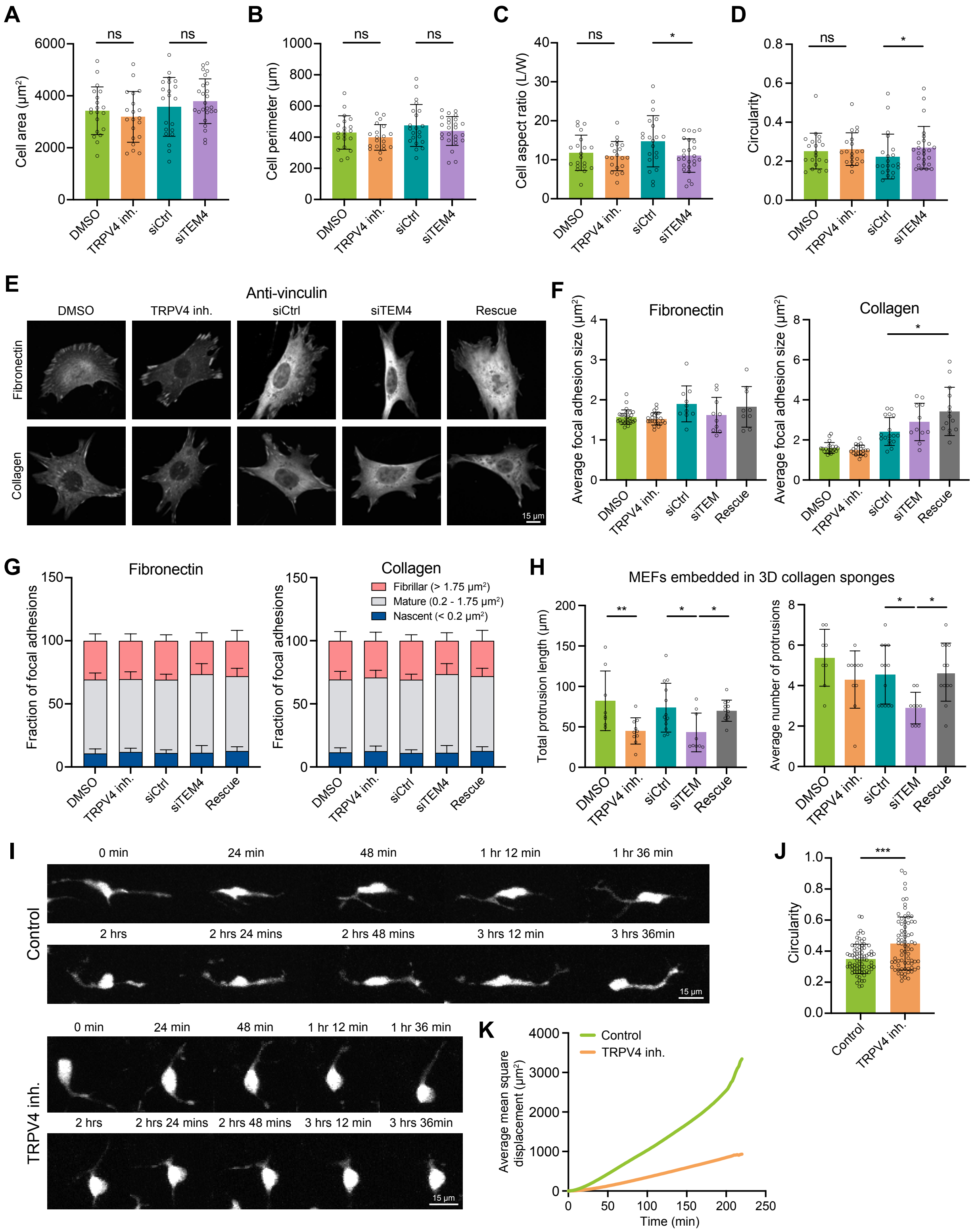

bioRxiv preprint doi: https://doi.org/10.1101/2025.03.28.646012; this version posted March 30, 2025. The copyright holder for this preprint (which was not certified by peer review) is the author/funder, who has granted bioRxiv a license to display the preprint in perpetuity. It is made available under aCC-BY 4.0 International license.

Figure S7. Suppression of TRPV4 signaling axis reduces cell protrusive activity while having a minimal effect on cell-ECM interaction and cell morphology. Related to Figure 7.

(A-D) Cell morphological analysis of MEFs plated on a FN-coated coverslips upon TRPV4 inhibition or TEM4 depletion. $n \geq 20$ cells per condition.

(E-G) Analysis of the effects of TRPV4 inhibition and TEM4 depletion on focal adhesions. (E) Fluorescence images of MEFs stained for vinculin. Cells were treated with 0.1% DMSO, TRPV4 inhibitor (10 µM HC-067047), depleted for TEM4 or TEM4-rescued. Cells were plated on (top) fibronectin- or (bottom) collagen-coated glass coverslips. (F) Quantifications of the average focal adhesion size per cell on (left) fibronectin- and (right) collagen-coated glass coverslips. (G) Distribution of focal adhesion size (left) on fibronectin- and (right) collagen-coated substrates. $n \geq 10$ cells per condition.

(H) Quantifications of (left) the total protrusion length and (right) the average number of protrusions of MEFs embedded in a 3D collagen matrix. $n \geq 10$ cells per condition.

(I-K) Morphological and migration analysis of melanoblasts. Melanoblasts were imaged in *Mitf-Cre::Rosa26-floxed stop-tdTomato* E15.5 mouse embryo. (I) Timelapse images of melanoblast cell expressing tdTomato in (top) control and (bottom) TRPV4 inhibition. (J) Quantification of melanoblast cell circularity in the presence of TRPV4 inhibitor. (K) Quantification of the average mean square displacement (MSD) upon TRPV4 inhibition. $n \geq 74$ cells per condition.

Mann-Whitney test is used for comparisons between 2 treatments and one-way ANOVA followed by Tukey-Kramer post-hoc test for comparisons between 3 or more treatments. The data are presented as mean ± SD unless otherwise specified. * $p < 0.05$, ** $p < 0.01$, *** $p < 0.005$, **** $p < 0.001$.

bioRxiv preprint doi: https://doi.org/10.1101/2025.03.28.646012; this version posted March 30, 2025. The copyright holder for this preprint (which was not certified by peer review) is the author/funder, who has granted bioRxiv a license to display the preprint in perpetuity. It is made available under aCC-BY 4.0 International license.

SUPPLEMENTAL VIDEO LEGENDS

Video S1. Lamellipodial protrusion upon TRPV4 suppression. Related to Figure 1.

MEFs transfected with siCtrl, siTRPV4 or siTRPV4 and eGFP-hTRPV4 (siRNA resistant TRPV4) migrating on 2.5 µg/mL FN-coated coverslips. DIC images were captured every 3 sec for 3 min 45 sec at 37°C. Top panels: Zoom-in views of a lamellipodial protrusion; Bottom panel: The entire migrating MEFs. The yellow box indicates where the zoom-in views was selected.

Video S2. Calcium changes in lamellipodia before and after TRPV4 inhibition. Related to Figure 2.

MEFs transfected with mCherry-T2A-GCaMP6f and F-tractin-emiRFP670 migrating on 2.5 µg/mL FN-coated coverslips. Multi-color fluorescence images were captured every 10 sec for 5 min 40 sec at 37°C. The cells were cultured in 0.1% DMSO supplemented phenol-red free DMEM in the first 1 min 50 sec and in 10 µM TRPV4 inh. supplemented phenol-red free DMEM after 2 min. Left panel: Timelapse images of ratiometric GCaMP6f/mCherry. Warmer colors indicate a higher Ca^{2+} level; Right panel: Timelapse images of F-tractin-emiRFP670 of a lamellipodial protrusion.

Video S3. NP-EGTA-AM-induced lamellipodial protrusion. Related to Figure 2.

MEFs transfected with mCherry-T2A-GCaMP6f and F-tractin-emiRFP670 migrating on 2.5 µg/mL FN-coated coverslips. Multi-color fluorescence images were captured every 15 sec for 30 sec before Ca^{2+} uncaging and for 2 min after the uncaging. The white box indicates the region of 405 nm photo-illumination. Top panels: F-tractin-emiRFP670 images of MEFs; Bottom panels: GCaMP6f images of MEFs. Brighter colors in the lookup table indicate a higher Ca^{2+} level; Left and Right panels: MEFs without and with NP-EGTA-AM.

Video S4. Optogenetic inhibition of CaMKII in lamellipodia. Related to Figure 5.

MEFs transfected with histone H2B-eBFP2 (Left; (-) paAIP2) or histone H2B-eBFP2-P2A-paAIP2 (Right; (+) paAIP2) were seeded in 2.5 µg/mL FN-coated glass-bottom dishes. Timelapse DIC images were acquired every 3 sec for 36 sec before and 5 min after 405 nm photo-illumination (3 sec). The purple box indicates the region of photo-illumination.

Video S5. Effects of TRPV4 inhibition and TEM4 depletion on 2D migration. Related to Figure 7.

MEFs transfected with histone H2B-mEmerald migrating on 2.5 µg/mL FN-coated glass coverslips. Top panels: MEFs treated with 0.1% DMSO or 10 µM TRPV4 inh. Bottom panels: MEFs co-transfected with siCtrl or siTEM4. For each condition, timelapse DIC and fluorescent histone H2B images were acquired every 12 min. The color code on the histone H2B timelapse images represents the time.

Video S6. Effects of TRPV4 inhibition on melanoblast migration. Related to Figure 7.

Melanoblast migration in E15.5 trunk skin explants (left) without or (right) with 10 µM TRPV4 inh. To label the embryonic melanoblasts, a melanocyte-specific *Mitf-Cre* transgene was used to induce tdTomato expression from a *Rosa26-floxed stop-tdTomato* fluorescent reporter. Confocal z-stacks were taken every 2 min continuously for 4 hr.
